# Contrast clearance analysis in neuro-oncology: A systematic review and meta-analysis on differentiating posttreatment changes from tumor progression

**DOI:** 10.1093/noajnl/vdaf161

**Published:** 2025-07-19

**Authors:** Mohammadreza Tahamtan, Mahshad Afsharzadeh, Masoumeh Sarvari, Shahryar Rahmani, Mahsa Geravandi, Delaram Amiri, Shahriar Kolahi

**Affiliations:** Advanced Diagnostic and Interventional Radiology Research Center (ADIR), Department of Radiology, Imam Khomeini Hospital, Tehran University of Medical Sciences, Tehran, Iran; Isfahan Neurosciences Research Center, Isfahan University of Medical Sciences, Isfahan, Iran; Advanced Diagnostic and Interventional Radiology Research Center (ADIR), Department of Radiology, Imam Khomeini Hospital, Tehran University of Medical Sciences, Tehran, Iran; Advanced Diagnostic and Interventional Radiology Research Center (ADIR), Department of Radiology, Imam Khomeini Hospital, Tehran University of Medical Sciences, Tehran, Iran; Advanced Diagnostic and Interventional Radiology Research Center (ADIR), Department of Radiology, Imam Khomeini Hospital, Tehran University of Medical Sciences, Tehran, Iran; Department of Radiology, School of Medicine, Isfahan University of Medical Sciences, Isfahan, Iran; Advanced Diagnostic and Interventional Radiology Research Center (ADIR), Department of Radiology, Imam Khomeini Hospital, Tehran University of Medical Sciences, Tehran, Iran; Advanced Diagnostic and Interventional Radiology Research Center (ADIR), Department of Radiology, Imam Khomeini Hospital, Tehran University of Medical Sciences, Tehran, Iran

**Keywords:** brain tumors, Contrast Clearance Analysis, pseudoprogression, radiation necrosis, TRAMs

## Abstract

**Background:**

Differentiating tumor progression from posttreatment changes, such as pseudoprogression and radiation necrosis, remains a significant challenge in neuro-oncology. Contrast Clearance Analysis (CCA), or Treatment Response Assessment Maps, has developed as a promising tool for this purpose. This systematic review and meta-analysis evaluate the diagnostic accuracy of CCA in distinguishing tumor progression from treatment-induced changes and compare its performance with other advanced imaging modalities.

**Methods:**

Following PRISMA-DTA guidelines, a comprehensive search was conducted across PubMed, Scopus, Web of Science, and Embase up to May 2025. Quality assessment was performed using the QUADAS-2 tool. Diagnostic accuracy metrics, including sensitivity, specificity, and area under the curve (AUC), were pooled using a bivariate random-effects meta-analysis model.

**Results:**

Nine studies involving 240 patients and 407 brain lesions were included. Contrast Clearance Analysis demonstrated a pooled sensitivity of 91% (95% CI, 0.84-0.95), a specificity of 92% (95% CI, 0.87-0.95), and an AUC of 88%. Moderate heterogeneity was observed in specificity (*I*² = 37.3%), with no significant heterogeneity in sensitivity (*I*² = 0%). Publication bias was detected (*P* <.001), with the trim-and-fill method suggesting 5 potentially missing studies. Quality assessment revealed a considerable risk of bias in the reference test domain.

**Conclusion:**

Contrast Clearance Analysis demonstrates high diagnostic accuracy in differentiating tumor progression from posttreatment changes, outperforming conventional MRI and showing comparable or superior performance to other advanced imaging techniques such as MR perfusion, diffusion-weighted imaging, and MR spectroscopy. However, methodological limitations and variability in reference standards highlight the need for standardized protocols in future research.

**Key Points**

- Contrast Clearance Analysis demonstrates a pooled sensitivity of 91% and specificity of 92% in differentiating tumor progression from posttreatment changes, outperforming conventional MRI and showing comparable or superior performance to other advanced imaging techniques.
- Contrast Clearance Analysis’s ease of acquisition and high diagnostic performance support its potential integration into routine clinical practice for treatment response assessment in brain tumor patients.
- Variability in reference standards (eg, histopathology vs clinic-radiological follow-up) and a focus on equivocal imaging cases in some studies highlight the need for standardized protocols and broader generalizability in future research.

**Importance of the Study**

This study systematically evaluates the diagnostic accuracy of Contrast Clearance Analysis (CCA) or Treatment Response Assessment Maps in differentiating tumor progression from treatment-related changes in brain tumors. While conventional MRI and advanced techniques such as perfusion-weighted imaging and MR spectroscopy have limitations in specificity and accessibility, CCA offers a high-resolution, relatively simple approach with superior sensitivity (91%) and specificity (92%). Our findings suggest that CCA may outperform existing modalities, providing a practical and widely applicable tool for clinical decision-making. By addressing a critical diagnostic challenge in neuro-oncology, this study supports the integration of CCA into routine practice, potentially reducing unnecessary interventions and optimizing treatment strategies. Future research should focus on multicenter validation and standardization of CCA protocols to further establish its clinical utility.

Brain tumors, both primary and metastatic, are frequently treated with multimodal therapeutic strategies, including surgical resection, radiotherapy, and systemic therapies. However, interpreting posttreatment imaging remains a significant clinical challenge, as conventional imaging techniques often cannot reliably differentiate between true tumor progression and treatment-related changes, such as pseudoprogression or radiation necrosis. Pseudoprogression, commonly observed in glioblastoma patients receiving concurrent radiotherapy and chemotherapy, presents as a transient increase in contrast enhancement on imaging, resembling tumor progression. However, unlike true progression, these changes typically stabilize or resolve over time without indicating disease. Conversely, radiation necrosis represents irreversible tissue damage following radiation therapy and may present similar imaging characteristics to recurrent or residual tumor, further complicating clinical decision-making.^1,2^ Conventional anatomical MRI sequences, including contrast-enhanced T1-weighted imaging and T2-weighted fluid-attenuated inversion recovery (FLAIR), lack sufficient specificity to differentiate viable tumor tissue from treatment-induced alterations reliably. This diagnostic ambiguity has important clinical implications, affecting treatment strategies and potentially leading to unnecessary procedures or delays in modifying therapy.^3^ Thus, advanced imaging modalities have been extensively considered to improve diagnostic accuracy in this critical setting. Advanced imaging modalities have been investigated to overcome the limitations of conventional MRI in differentiating true tumor progression from pseudoprogression or radiation necrosis. Magnetic resonance spectroscopy (MRS) offers high sensitivity in detecting tumor recurrence; however, its limited spatial resolution and overlapping metabolic signatures reduce its diagnostic specificity.^4^ Perfusion-weighted imaging evaluates tumor vascularity, utilizing dynamic susceptibility contrast (DSC) MRI to measure relative cerebral blood volume (rCBV). This technique helps differentiate recurrent tumors, which typically show elevated rCBV, from treatment-related changes, which usually exhibit lower rCBV. However, variations in rCBV thresholds and susceptibility artifacts can limit the accuracy of this imaging method. Dynamic contrast-enhanced (DCE) MRI evaluates vascular permeability via K trans values, with increased K trans suggesting viable tumor tissue.^5,6^ Diffusion-weighted imaging (DWI) characterizes tissue microstructure, where tumor recurrence typically exhibits restricted diffusion due to high cellular density, whereas treatment-related changes demonstrate increased diffusivity. However, variations in apparent diffusion coefficient values among different tumor types and treatment protocols reduce the reliability of diffusion-based techniques for assessing treatment response.^7^

Contrast Clearance Analysis (CCA), also known as Treatment Response Assessment Maps (TRAMs), has emerged as a novel approach for differentiating tumoral progression from posttreatment changes. This technique relies on the delayed clearance or retention of contrast agents within lesions. Unlike conventional perfusion MRI, which focuses on first-pass contrast kinetics, CCA involves taking delayed postcontrast T1-weighted images, usually 60-90 min after injection, to evaluate differences in contrast agent clearance. Viable tumor tissue, characterized by high vascular permeability and rapid contrast washout, is typically represented as a blue signal on TRAMs, whereas areas of contrast accumulation (red signal) suggest radiation necrosis or treatment-related changes.^8^

Recent studies have demonstrated the potential of CCA/TRAMs in improving diagnostic accuracy compared with conventional MRI and other advanced imaging techniques. By offering a model-independent and high-resolution approach to tissue differentiation, CCA has shown promise in guiding clinical decision-making and optimizing treatment strategies for brain tumor patients.^8,9^ However, more systematic validation is needed to confirm its utility in routine neuro-oncologic imaging.

This systematic review and meta-analysis aim to evaluate the diagnostic accuracy of CCA or TRAMs, in differentiating tumor progression from treatment-related changes. In addition, we contextualize its performance by comparing our findings with those reported in existing systematic reviews and meta-analyses of other advanced imaging modalities, such as MRS, DCE and DSC perfusion MRI, and diffusion-based imaging, to assess its potential clinical utility.

## Methods

The present study has been officially registered in the International Prospective Register of Systematic Reviews (PROSPERO) under the registration number CRD42024623509. The document follows the Preferred Reporting Items for Systematic Reviews and Meta-Analyses of Diagnostic Test Accuracy Studies (PRISMA-DTA) guidelines, as cited in references.^10,11^

### Search method

A comprehensively systematic search was conducted employing four major electronic medical literature databases: These databases comprise PubMed, Scopus, Web of Science, and Embase. The search criteria were developed based on keywords associated with “Pseudo-progression,” “recurrence,” “Brain Tumor,” “magnetic resonance imaging,” and “follow-up.” Publications up to May 2025 were included. The search strategies for each database are documented in the supplementary file.

The findings were exported to Endnote Desktop software to simplify the organization and duplicate entries elimination. Review Manager (RevMan) (computer program), Version 5.4, developed by The Cochrane Collaboration in 2020, was utilized for the management of data relevant to the systematic review. To ensure a comprehensive review, a manual review of the reference lists of the full-text articles obtained was also conducted to ascertain that no additional studies were overlooked.

### Study selection

Endnote Desktop was employed to screen the abstracts and titles of the articles. Two independent researchers, M.A. and M.S., who were blind to each other’s selections, evaluated which studies met the inclusion criteria. After the preliminary assessment, M.T. examined the selections made by M.A. and M.S., and they resolved any discrepancies through discussion, ultimately reaching a final consensus on which studies to include. The inclusion criteria targeted original research articles that assessed the diagnostic capabilities of CCA for differentiating pseudo-progression from tumor recurrence following brain tumor treatment. Otherwise, the exclusion criteria consisted of unpublished manuscripts, case reports, animal studies, conference materials, articles in languages other than English, and articles which the full text was unavailable or data necessary for constructing a 2 × 2 contingency table was not provided. In 1 study, CCA results were classified into percentages reflecting radiation necrosis and tumoral progression based on histopathological evaluation. Each lesion was categorized according to the higher percentage it showed between the 2 conditions.

### Extraction of data and assessment of quality

The extraction and assessment process were conducted independently by 2 authors, M.A. and M.T., who conducted a detailed review of the full texts of all selected articles. Both qualitative and quantitative data were extracted from each study. The information they collected included: (1) General details of the studies, including the title, publication year, first author, country, and study design. (2) Participants’ characteristics, such as the total number of participants, patients’ demographics, types of MRI sequences, contrast dose, time of delay, tumor histology, treatment details, standard of reference, and interval between MRI and reference standard. (3) Outcome parameters, including numerical data on patients with post-treatment brain tumors required to construct a 2 × 2 contingency table. For quantitative analysis, metrics such as sensitivity, specificity, true positives (T.P.), false positives (F.P.), true negatives (T.N.), and false negatives (F.N.) were extracted from the data and entered into RevMan software to facilitate synthesis.

The QUADAS-2 instrument, a 17-item tool standardized for the assessment of methodological quality in systematic reviews of diagnostic test accuracy,^12^ was used to evaluate the quality of the studies. The QUADAS-2 tool is categorized into 4 domains: patient selection, risk of bias and applicability of the index test, risk of bias and applicability of the reference standard test, and the flow and timing of the study. Responses to these questions should be classified as “yes,” “no,” or “unclear.” For the present analysis, the QUADAS-2 functionality in Review Manager was employed to evaluate the methodological quality of the papers included.

### Statistical analysis

To perform a diagnostic test accuracy (DTA) meta-analysis, spreadsheets containing TP, FP, FN, and TN values were retrieved from RevMan for quantitative analysis using suitable models. The bivariate random-effects meta-analysis model was applied to generate summary receiver operating characteristic (SROC) curves.^13^ The heterogeneity of the data was evaluated both visually and using the I2 statistics proposed by Zhou and Dendukuri.^14^ The presence of publication bias in the DTA meta-analysis was examined using Deek’s funnel plot asymmetry test, with a *P*-value below .10 being suggestive of significant bias. The analyses were conducted using the MADA and META custom modules in R project.^15,16^

## Results

### Study characteristics

**Figure 1:**
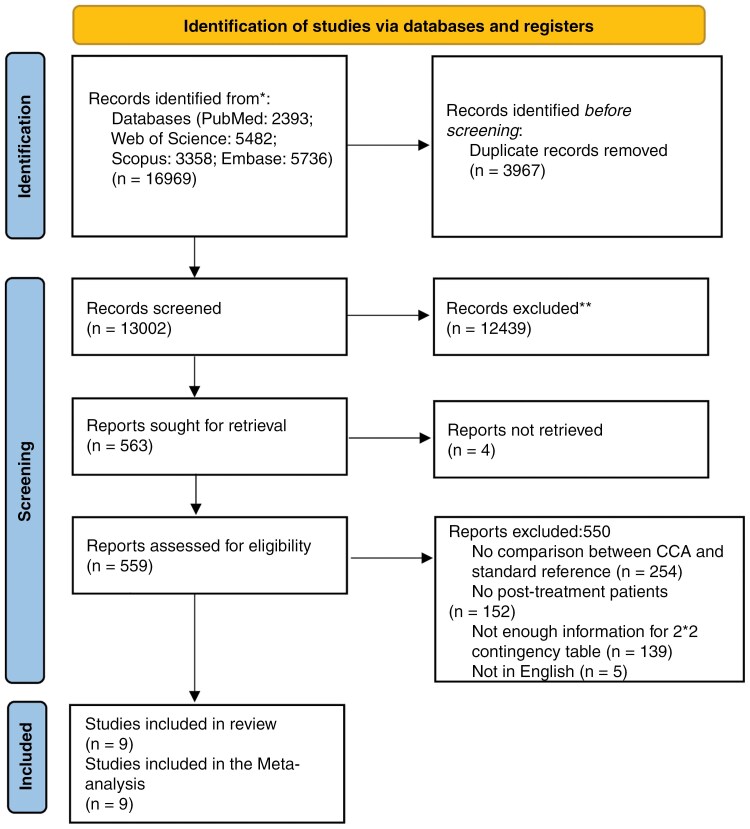
PRISMA flow diagram shows the study selection process.

During our search across 4 online databases, a total of 13,002 studies were identified after deduplication. These were initially screened based on titles and abstracts, resulting in the exclusion of 124,391 articles. Of the remaining 563 studies, 4 were inaccessible in full text. A comprehensive review of the remaining 559 full-text articles led to the exclusion of 550 studies due to irrelevance to the research question, insufficient data for a 2 × 2 contingency table, foreign language, or because they were review articles. Finally, 9 studies were selected for inclusion in the systematic review and meta-analysis. The process of exclusions and the rationale behind them are presented in the PRISMA flow diagram^17^ (Figure 1).

A total of 9 studies were included in the meta-analysis, including 240 patients and 407 brain lesions. Among these patients, 106 (44.2 %) were female and 134 (55.8 %) were male. Histopathological assessment was the reference standard across 3 studies.^4,5,18^ However, most of the studies also used a combination of histopathology, clinical evaluation, and imaging as reference criteria. Moreover, 2 studies relied on clinicoradiological follow-up as the reference standard.^8,19^ The overall mean age was 57.57 ± 11.68 years. Four studies were prospective,^3,5,19,20^ while the remaining 4 were retrospective. The included studies reported various tumor histologies, including primary brain tumors, such as glioblastoma, anaplastic astrocytoma, and low-grade glioma, as well as secondary brain tumors. The most common metastatic brain lesions originated from the lung, breast, and melanoma. The reviewed studies encompassed various therapeutic strategies for brain tumors, incorporating advanced radiotherapy techniques, systemic treatments, and surgical methods. The radiotherapy techniques varied widely, including stereotactic radiosurgery (SRS), whole-brain radiotherapy (WBRT), and partial-brain radiotherapy, administered through both hypofractionated and normofractionated dosing schedules. Systemic interventions primarily involved chemotherapy, delivered either concurrently or sequentially with other treatments. Surgical resection was employed either as an independent therapeutic measure or in combination with SRS and WBRT to enhance treatment efficacy.

Contrast Clearance Analysis was conducted in all included studies by subtracting delayed T1-weighted images from early T1 contrast-enhanced MRI scans. The delay times varied between 55 and 86 min across the studies. Additional details regarding gender, age, and other characteristics are provided in Table 1.

**Table 1.** Characteristics of the included studies.

Author, date	Country	Design	Tumor type	Field strength	Patient	Lesion	Age	Male (%)	Reference standard	time of delay (min)
Wagner 2017^19^	Germany	Prospective	Secondary brain tumor	3T	31	41	50.4 (33-71) (Mean, Range)	51.6	Clinicoradiologic follow-up	55
Zach 2012^20^	Israel	Prospective	Mixed	3T	20	40	54.4 ± 3.8 (Mean, SD)	65.0	Mixed	75
Admojo 2023^3^	Australia	Prospective	Secondary brain tumor	3T	10	10	52 (41-75) (Median, Range)	30.0	Mixed	62.5
Bodensohn 2022^5^	Germany	Prospective	Mixed	3T	33	33	NA	48.5	Histopathological	60
Lakehayli 2023^21^	France	Retrospective	Secondary brain tumor	NA	10	10	55.9 ± 10.3 (Mean, SD)	70	Mixed	75
Müller 2024^4^	Germany	Retrospective	Primary brain tumor	3-1.5T	27	29	59 ± 12 (Mean, SD)	55.6	Histopathological	86
Mohamedkhan 2024^7^	UK	Retrospective	Secondary brain tumor	NA	62	104	66 (33-90) (Mean, Range)	61.3	Mixed	60
Peker 2020^8^	Turkey	Retrospective	Secondary brain tumor	3T	37	130	58 (38-74) (Median, Range)	54.1	Clinicoradiologic follow-up	81.8
Alkhatatneh 2025^18^	USA	Retrospective	Mixed	NA	10	10	58 (16) (Mean, IQR)	60.0	Histopathological	82.5

### Quality assessment

**Figure 2:**
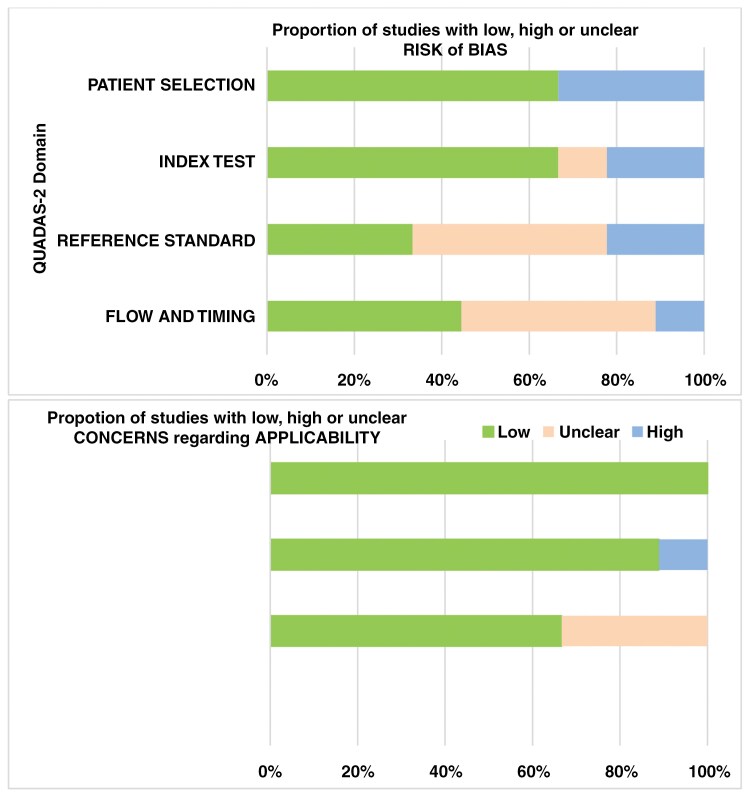
Risk of bias and applicability concerns summary for included studies using QUADAS-2 tool.

We employed the QUADAS-2 tool to assess the quality of the included studies, as demonstrated in Figure 2. Our analysis revealed a significant risk of bias across several domains, specifically, in the reference test domain. Only 3 studies^4,5,18^ used histopathological evaluation as the reference standard, while the remaining studies relied on mixed or clinic-radiological follow-up, which may introduce variability and reduce diagnostic certainty.

Additionally, in the patient selection domain, 3 studies^3,5,21^ included patients with equivocal brain imaging findings, raising concerns about the generalizability and reliability of their results. In the patient flow domain, only 1 study demonstrated a high risk of bias, as it applied different reference standards and variable follow-up durations to patients, potentially compromising the consistency and validity of its findings. These methodological limitations highlight the need for cautious interpretation of the results and highlight the importance of standardized protocols in future research.

### Meta-analysis results

**Figure 3:**
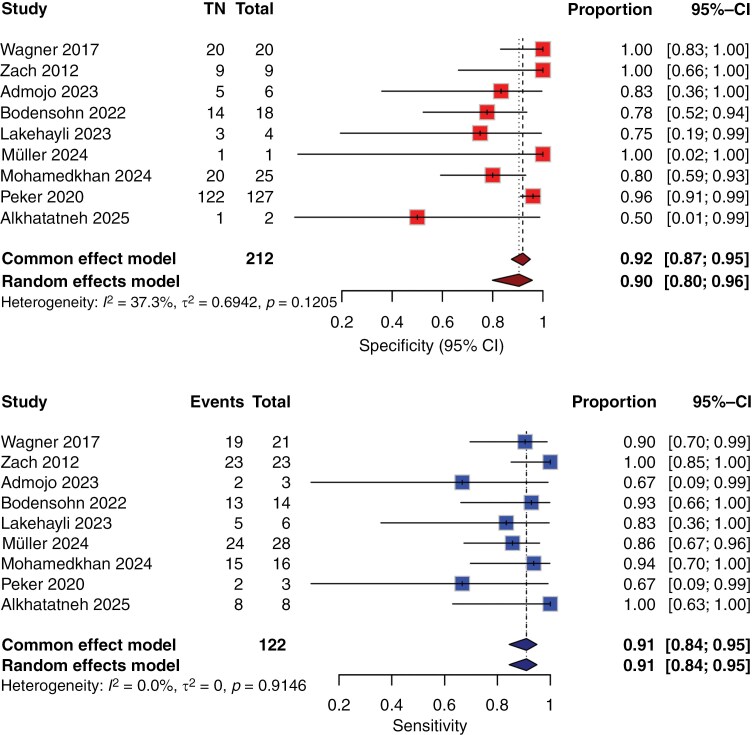
Paired forest plots of sensitivity and specificity of random effects bivariate-model meta-analysis of diagnostic accuracy of contrast clearance study in detecting recurrence from posttreatment changes. CI: Confidence interval.

**Figure 4:**
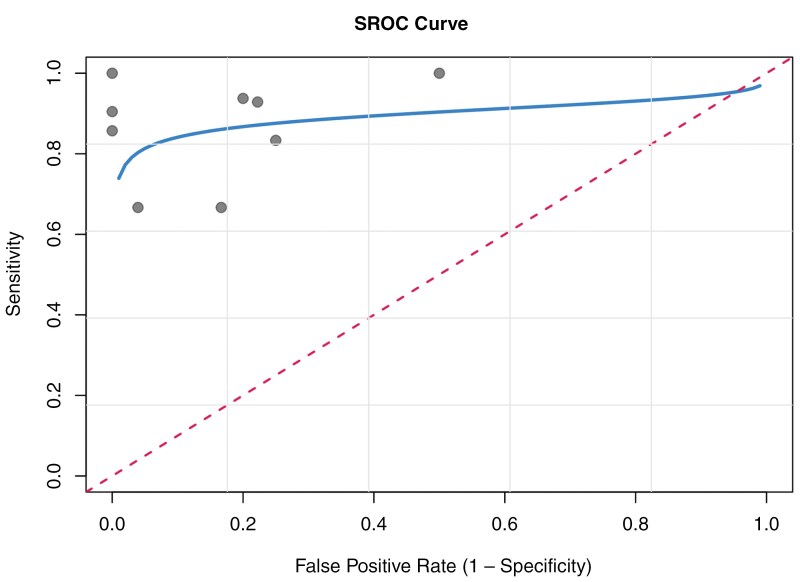
Smoothed receiver operating characteristic (SROC) curve for CCA diagnostic accuracy. The solid line represents the SROC curve. Circles indicate individual study points, each corresponding to a unique study. The diagonal dashed line represents the reference line for random chance.

In the meta-analysis encompassing all included studies, the pooled sensitivity was determined to be 91% (95% CI, 0.84-0.95), accompanied by a pooled specificity of 92% (95% CI, 0.87-0.95) and an area under the curve (AUC) of 88%, as depicted in Figure 3. The bivariate diagnostic random-effects meta-analysis showed moderate heterogeneity in specificity (*I*² = 37.3%), while no heterogeneity in sensitivity (*I*² = 0%). The *P*-value of Q test was greater than .001, suggesting no statistically significant heterogeneity. The diagnostic odds ratio (DOR) was estimated at 31.56 (95% CI, 13.31-74.83). The SROC curve analysis showed an AUC of 0.88, indicating high diagnostic accuracy (Figure 4).

### Publication bias

**Figure 5:**
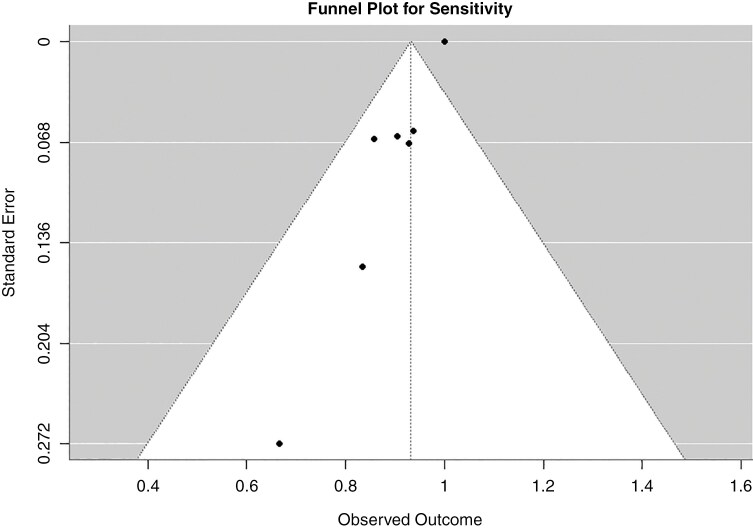
The funnel plot shows publication bias. The plot reveals statistically significant (*P* <.001) asymmetry, suggesting the presence of publication bias in the included studies.

Funnel plot asymmetry analysis showed significant publication bias (*P* <.001). The trim-and-fill method estimated that 5 missing studies were likely on the right side of the funnel plot (Figure 5).

## Discussion

Differentiating tumoral progression or recurrence from posttreatment changes remains a critical challenge in managing brain and head and neck malignancies. These malignancies often require multimodal treatment, including surgery, chemotherapy, and radiotherapy. Accurately distinguishing these entities is essential, as it directly impacts treatment planning and follow-up strategies. However, this differentiation remains a diagnostic dilemma.

Various MRI techniques have been explored to resolve this challenge. Among them, MR perfusion studies, DWI, and MRS are well-established methods, with multimodal MRI approaches increasingly employed to enhance diagnostic accuracy. Recently, CCA, also known as the TRAMs, has gained attention as an alternative imaging technique. This method has been evaluated in various experimental and clinical settings, with promising reports regarding its sensitivity and specificity.

Our meta-analysis demonstrated that CCA achieves a sensitivity of 91% and a specificity of 92%, with an AUC of 88%, indicating high diagnostic accuracy in differentiating tumoral progression from posttreatment changes. Contrast Clearance Analysis is a postcontrast imaging technique that involves acquiring T1-weighted images 5 min after contrast injection and subtracting them from delayed T1-weighted images obtained 60-90 min postinjection.^5^ The underlying principle relies on the contrast washout pattern: in posttreatment changes, impaired vascular integrity and blood–brain barrier disruption result in prolonged contrast retention, whereas in viable tumor tissue, contrast washout is observed. This allows for the characterization of tissue based on contrast clearance dynamics.^5,7^ Zach et al. conducted one of the earliest CCA in posttreatment malignant brain tumors, linking rapid and slow clearance regions identified in imaging with histopathologic findings. Their study revealed a strong relationship between the imaging-based areas of tumor or necrosis and corresponding histopathological results. They classified a lesion as tumoral if the majority of the lesion exhibited rapid clearance, while lesions were deemed posttreatment changes if they were predominantly slow clearance.^20^ However, this approach appears overly simplistic, and interpreting enhancement and clearance patterns may offer a more precise method for assessing lesion status compared with relying solely on the percentage of red versus blue regions. Admojo et al. evaluated lesions by analyzing peripheral rim and interior enhancement and clearance patterns, demonstrating that this method achieves greater accuracy in lesion interpretation compared with the approach proposed by Zach et al.^3^

Bodensohn et al. reported CCA’s high diagnostic accuracy in distinguishing radiation-induced changes from tumoral progression after cranial radiotherapy, with a sensitivity of 93% and a specificity of 78%.^5^ They mentioned that CCA has the potential to delay or even eliminate the need for biopsies in ambiguous cases. Compared with most diagnostic approaches, CCA offers a significant advantage: it relies solely on an MRI scanner, making it a widely accessible method for distinguishing pseudoprogression from true tumor progression in most patients.

A significant advantage of using the delayed enhancement and clearance rates instead of the commonly studied early rates (DCE and DSC) is the ability to apply sequences with lower temporal resolution, such as high resolution spin-echo T1-MRI. These sequences nearly completely avoid susceptibility artifacts while providing high signal-to-noise ratios, high resolution and high sensitivity to contrast variations. In the other hand, lower temporal resolution could be a pitfall in smaller lesions (<5mm).^5,20^ Another challenge in CCA lies in determining a threshold for washout. Cuccarinini et al. addressed this by defining a threshold based on the ratio of washed-out regions (depicted as blue on TRAM maps) to the primary volume of contrast enhancement. Through receiver operating characteristic analysis, they determined a VBlue/VCE variation threshold of −0.066, which achieved 71.4% sensitivity and 100% specificity in distinguishing true progression from pseudoprogression.^22^

Similarly, Mohamedkhan et al. demonstrated that CCA had a significant impact on treatment decisions in cases with equivocal MRI findings after SRS for brain metastases, reinforcing its clinical utility.^7^

Compared with MR perfusion techniques such as DSC, DCE imaging, and arterial spin labeling (ASL), CCA presents a distinct advantage. Unlike DSC, DCE, and ASL, which rely on the first-pass effect for perfusion assessment, CCA is based on contrast washout dynamics.^23,24^ A systematic review and meta-analysis by Teunissen et al. reported that DSC achieved a sensitivity of 83% and a specificity of 78%, while DCE yielded 74% sensitivity and 92% specificity.^2^ Similarly, Smith et al. reported DSC sensitivity and specificity of 85% and 86%, respectively, and DCE sensitivity and specificity of 85% and 78%.^1^ In comparison, our findings for CCA (91% sensitivity and 92% specificity) suggest a superior sensitivity relative to both DSC and DCE. However, it is important to note that all 3 methods require contrast administration, and CCA involves an additional delayed imaging step (60-90 min postinjection), which is not necessary for DSC or DCE.

One advantage of CCA over DSC is its resistance to susceptibility artifacts, as it relies on T1-weighted imaging rather than the T2*-weighted sequences used in DSC. Moreover, study by Lakehayli et al. have suggested that while DCE perfusion MRI has strong performance in differentiating radionecrosis from recurrence, CCA should be integrated into routine practice to complement perfusion imaging.^21^

Moreover, while ASL offers a noncontrast alternative, studies evaluating its diagnostic performance remain limited. Lai et al. reported ASL sensitivity and specificity of 83% and 100%, respectively, for differentiating tumor recurrence from necrosis in brain metastases following stereotactic radiosurgery, with an accuracy of 92%.^25^ Smith et al., in their meta-analysis, reported an ASL sensitivity and specificity of 86% and 84%, respectively,^1^ which are both lower than the values we found for CCA in our meta-analysis. In contrast, our meta-analysis revealed CCA’s sensitivity and specificity of 91% and 92%, respectively, with an accuracy of 88%. Despite ASL’s noncontrast nature, its clinical adoption is hindered by acquisition and interpretation complexities, a steep learning curve, and limited availability across MRI platforms.

Magnetic resonance spectroscopy has also been used for this purpose. According to Teunissen et al., MRS demonstrates a sensitivity and specificity of 80% and 78%, respectively,^2^ while Smith et al. reported 90% sensitivity and 84% specificity.^1^ Although these values are comparable to those of CCA and despite its strong performance, MRS has technical limitations, including long acquisition time, complex post-processing, and challenges in spectral interpretation, particularly in heterogeneous lesions.^26^ In contrast, CCA provides a simpler and more standardized approach with high reproducibility.

Diffusion-weighted imaging has also been investigated for distinguishing posttreatment changes from tumoral progression. Teunissen et al. reported a sensitivity of 67% and a specificity of 79% for DWI,^2^ while Smith et al. found values of 81% and 78%, respectively.^1^ Both studies indicate that DWI has lower diagnostic performance compared with CCA. However, DWI and CCA are fundamentally different modalities, with DWI relying on the diffusion properties of water molecules rather than contrast kinetics.

Positron emission tomography (PET) and single-photon emission computed tomography (SPECT) have also been employed for this differentiation, leveraging metabolic imaging to assess tissue activity. Smith et al. reported that 18F-DOPA PET achieved a pooled sensitivity of 89.8% and a specificity of 88.0%, while 11C-CHO PET demonstrated a sensitivity and specificity of 87.2% and 88.4%, respectively. For SPECT, the pooled sensitivity and specificity were reported as 88.7% and 88.3%, respectively.^1^ Although these values are similar to those of CCA, PET, and SPECT are limited by acquisition complexity, high costs, and limited availability, reducing their feasibility for routine clinical use.

The findings of this meta-analysis highlight the clinical relevance of CCA as a noninvasive imaging modality with high diagnostic accuracy for treatment response assessment. TRAMs are highly effective in differentiating posttreatment changes from tumor progression across a broad spectrum of brain lesions. Kowa et al. evaluated the diagnostic performance of TRAMs and 18F-Choline PET at different disease stages in a patient with primary CNS lymphoma. Their findings indicated that both modalities were superior to contrast-enhanced MRI for differentiating postbiopsy changes and treatment response from active tumor tissue, as well as for confirming complete response, especially in scenarios where a few enhancing lesions persisted.^25^ Moreover, TRAMs showed comparable advantages in a biopsy-confirmed case of primary CNS lymphoma with a callosal lesion. “End-of-treatment” TRAMs demonstrated no evidence of active disease at the site of MRI enhancement. This application of TRAMs eliminated the need for consolidation radiotherapy in this case.^27^

TRAM-based assessments effectively differentiated between radiation-induced changes and persistent tumoral lesions in metastatic brain tumors treated with Gamma Knife radiosurgery, reinforcing its potential in neuro-oncologic practice.^8^ Additionally, Müller et al. highlighted that TRAMs improved diagnostic confidence when distinguishing recurrent glioblastoma from radiation necrosis, further supporting its clinical application.^4^ TRAMs provide a valuable tool for distinguishing true tumor progression from pseudoprogression in IDH wild-type glioblastoma patients undergoing radiotherapy, temozolomide therapy, and even immunotherapy.

## Limitations

This meta-analysis has several important limitations. First, the available studies on CCA are limited in number and exhibit considerable heterogeneity in both tumor pathology and reference standards. While some studies use histopathological confirmation, others rely on clinicoradiological follow-up as the diagnostic gold standard, introducing variability in outcome definitions. Second, the potential for publication bias cannot be excluded, particularly given the absence of studies reporting negative or inconclusive results, which may overestimate the diagnostic accuracy of CCA. Third, none of the included studies reported lesion size, precluding analysis of its potential impact on diagnostic performance. Additionally, the inclusion of small case series may introduce bias and influence the pooled estimates. Lastly, the wide confidence interval of the DOR is likely due to small sample sizes and near-perfect accuracy in some studies. The results should be interpreted cautiously, as the pooled estimates may not reliably reflect true diagnostic performance. These limitations highlight the need for future large-scale, standardized studies to better evaluate and validate the clinical utility of CCA.

## Conclusion

This meta-analysis comprehensively evaluates the diagnostic performance of CCA across existing literature, highlighting its ease of acquisition and analysis. The results of this systematic review should be interpreted with caution due to the potential presence of publication bias, which may lead to an overestimation of the effectiveness or accuracy of the evaluated approaches. However, the absence of significant heterogeneity in our findings strengthens the consistency and reliability of the results. With a sensitivity of 91%, specificity of 92%, and AUC of 88%, CCA presents a promising addition to the imaging toolbox for distinguishing tumoral progression from posttreatment changes. Its high diagnostic accuracy, along with its relative simplicity compared with other imaging modalities, supports its potential role in clinical decision-making.

## Supplementary Material

vdaf161_suppl_Supplementary_Materials_1

## Data Availability

No new data were generated or analyzed in support of this research. All data used in this study are available from the original studies cited in the references.
