# Supplementary material for: Contrast clearance analysis in neuro-oncology: A systematic review and meta-analysis on differentiating posttreatment changes from tumor progression
Source: Neurooncol Adv. 2025 Jul 19;7(1):vdaf161. doi: 10.1093/noajnl/vdaf161 (PMC12311927; doi:10.1093/noajnl/vdaf161)
Supplement: vdaf161_suppl_Supplementary_Materials_1 [file vdaf161_suppl_supplementary_materials_1.docx]

Pubmed:

("Brain Neoplasms/radiotherapy"[Mesh] OR "Neoplasm Recurrence, Local"[Mesh] OR "Brain Neoplasms"[Mesh] OR "Glioma"[Mesh] OR "Astrocytoma"[Mesh] OR "Oligodendroglioma"[Mesh] OR "Glioblastoma"[Mesh] OR "Glioma, Subependymal"[Mesh] OR "Optic Nerve Glioma"[Mesh] OR "Neoplasm Metastasis/diagnosis"[Mesh] OR "Neoplasm Metastasis/diagnostic imaging"[Mesh] OR "Neoplasm Metastasis/radiotherapy"[Mesh] ) OR disease progress*[tiab] OR true progress*[tiab] OR recurrent glio*[tiab] OR residu*[tiab] OR recurr*[tiab] OR progress*[tiab] OR true tumo*[tiab] OR glioma*[tiab] OR (brain*[tiab] AND (tumor[tiab] OR tumour[tiab])) OR glioblastom[tiab] OR astrocytom*[tiab] OR oligodendrogliom*[tiab] OR oligoastrocytom*[tiab] OR (glia*[tiab] AND (tumor[tiab] OR tumour[tiab]) OR brain met*[tiab])

AND

(Abnormalities, Radiation-Induced/radiotherapy[Mesh] OR "Treatment Outcome"[Mesh] OR "radiation effects" [tiab] OR treatment-induc*[tiab] OR radiation induc*[tiab] OR radiation associat*[tiab] OR radiation chang*[tiab] OR radiation effect*[tiab] OR treatment necros*[tiab] OR chemoradiation*[tiab] OR treatment effect*[tiab] OR post treat*[tiab] OR posttreat*[tiab] OR posttherap*[tiab] OR post therap*[tiab] OR postsurg*[tiab] OR post-surg*[tiab] OR post irradiat*[tiab] OR postirradiat*[tiab] OR after irradia*[tiab] OR after rad*[tiab] OR post radiat*[tiab] OR postradiat*[tiab] OR treatment outcome*[tiab] OR radiation injur*[tiab] OR pseudo progress*[tiab] OR pseudo response OR pseudoprogress*[tiab] OR pseudorespon*[tiab] OR radiation necro*[tiab] OR radio necro*[tiab] OR radionecros*[tiab] OR treatment-relat*[tiab])

AND

("Magnetic Resonance Angiography"[Mesh] OR "MR perfusion"[tiab] OR MRI perfusion[tiab] OR perfusion-weighted MR[tiab] OR magnetic resonance perfusion[tiab] OR 'PWI'[tiab] OR "ASL"[tiab] OR arterial spin labeling[tiab] OR "DSC"[tiab] OR dynamic susceptibility contrast[tiab] OR "DCE"[tiab] OR Dynamic contrast enhanced [tiab] OR advanced MRI[tiab] OR multiparametric MR[tiab] OR Contrast enhanced MRI[tiab] OR contrast clearance[tiab] OR "TRAM" OR treatment response assessment*[tiab] OR delayed contrast MR[tiab] OR delayed contrast extravasation MR[tiab])

Embase:

('brain tumor'/exp/dm_rt OR 'tumor recurrence'/exp OR 'brain tumor'/exp OR 'glioma'/exp OR 'astrocytoma'/exp OR 'oligodendroglioma'/exp OR 'glioblastoma'/exp OR 'subependymoma'/exp OR 'optic nerve glioma'/exp OR 'metastasis'/exp/dm_di OR 'disease progress*':ti,ab,kw OR 'true progress*':ti,ab,kw OR 'recurrent glio*':ti,ab,kw OR 'residu*':ti,ab,kw OR 'recurr*':ti,ab,kw OR 'progress*':ti,ab,kw OR 'true tumo*':ti,ab,kw OR 'glioma*':ti,ab,kw OR ('brain*':ti,ab,kw AND ('tumor':ti,ab,kw OR 'tumour':ti,ab,kw)) OR 'glioblastom':ti,ab,kw OR 'astrocytom*':ti,ab,kw OR 'oligodendrogliom*':ti,ab,kw OR 'oligoastrocytom*':ti,ab,kw OR ('glia*':ti,ab,kw AND ('tumor':ti,ab,kw OR 'tumour':ti,ab,kw)) OR 'brain met*':ti,ab,kw)

AND

('radiation induced malformation'/exp/dm_rt OR 'treatment outcome'/exp OR 'radiation effects':ti,ab,kw OR 'treatment-induc*':ti,ab,kw OR 'radiation induc*':ti,ab,kw OR 'radiation associat*':ti,ab,kw OR 'radiation chang*':ti,ab,kw OR 'radiation effect*':ti,ab,kw OR 'treatment necros*':ti,ab,kw OR 'chemoradiation*':ti,ab,kw OR 'treatment effect*':ti,ab,kw OR 'post treat*':ti,ab,kw OR 'posttreat*':ti,ab,kw OR 'posttherap*':ti,ab,kw OR 'post therap*':ti,ab,kw OR 'postsurg*':ti,ab,kw OR 'post-surg*':ti,ab,kw OR 'post irradiat*':ti,ab,kw OR 'postirradiat*':ti,ab,kw OR 'after irradia*':ti,ab,kw OR 'after rad*':ti,ab,kw OR 'post radiat*':ti,ab,kw OR 'postradiat*':ti,ab,kw OR 'treatment outcome*':ti,ab,kw OR 'radiation injur*':ti,ab,kw OR 'pseudo progress*':ti,ab,kw OR 'pseudo response' OR 'pseudoprogress*':ti,ab,kw OR 'pseudorespon*':ti,ab,kw OR 'radiation necro*':ti,ab,kw OR 'radio necro*':ti,ab,kw OR 'radionecros*':ti,ab,kw OR 'treatment-relat*':ti,ab,kw)

AND

('magnetic resonance angiography'/exp OR 'mr perfusion':ti,ab,kw OR 'mri perfusion':ti,ab,kw OR 'perfusion-weighted mr':ti,ab,kw OR 'magnetic resonance perfusion':ti,ab,kw OR 'pwi':ti,ab,kw OR 'asl':ti,ab,kw OR 'arterial spin labeling':ti,ab,kw OR 'dsc':ti,ab,kw OR 'dynamic susceptibility contrast':ti,ab,kw OR 'dce':ti,ab,kw OR 'dynamic contrast enhanced':ti,ab,kw OR 'advanced mri':ti,ab,kw OR 'multiparametric mr':ti,ab,kw OR 'contrast enhanced mri':ti,ab,kw OR 'contrast clearance':ti,ab,kw OR 'tram' OR 'treatment response assessment*':ti,ab,kw OR 'delayed contrast mr':ti,ab,kw OR 'delayed contrast extravasation mr':ti,ab,kw)

Scopus:

( TITLE-ABS-KEY ( "brain neoplasms" OR "brain tumor" OR "glioma" OR "astrocytoma" OR "oligodendroglioma" OR "glioblastoma" OR "subependymal glioma" OR "optic nerve glioma" OR "brain metastasis" OR "local neoplasm recurrence" OR ( disease AND progress* ) OR ( true AND progress* ) OR ( recurrent AND glio* ) OR residu* OR recurr* OR progress* OR ( true AND tumo* ) OR glioma* OR ( brain* AND ( tumor OR tumour ) ) OR glioblastom* OR astrocytom* OR oligodendrogliom* OR oligoastrocytom* OR ( glia* AND ( tumor OR tumour ) ) OR brain AND met* ) )

AND

( TITLE-ABS-KEY ( "radiation injury" OR "radiation therapy" OR "treatment outcome" OR "radiation-induced disorder" OR "radiation necrosis" OR "chemoradiotherapy" OR "postoperative complication" OR "treatment related" OR ( radiation AND effect* ) OR ( treatment AND necros* ) OR chemoradiation* OR ( treatment AND effect* ) OR ( post AND treatment ) OR posttreatment OR ( post AND therapy ) OR posttherapy OR ( post AND surgery ) OR "post-surgical" OR ( post AND irradiation ) OR postirradiation OR ( after AND irradiation ) OR ( after AND radiation ) OR ( post AND radiation ) OR postradiation OR ( treatment AND outcome* ) OR ( radiation AND change* ) OR ( radiation AND associated ) OR "pseudo-progression" OR pseudoprogression OR pseudoresponse OR radionecrosis ) )

AND

( TITLE-ABS-KEY ( "magnetic resonance perfusion" OR "dynamic contrast enhanced MRI" OR "arterial spin labeling" OR "multiparametric MRI" OR "MR perfusion" OR "MRI perfusion" OR "perfusion-weighted MR" OR "magnetic resonance perfusion" OR "PWI" OR "ASL" OR "arterial spin labeling" OR "DSC" OR "dynamic susceptibility contrast" OR "DCE" OR "dynamic contrast enhanced" OR "advanced MRI" OR "multiparametric MR" OR "contrast enhanced MRI" OR "contrast clearance" OR "TRAM" OR "treatment response assessment" OR "delayed contrast MR" OR "delayed contrast extravasation MR" ) ) AND ( LIMIT-TO ( SUBJAREA , "MEDI" ) ) AND ( LIMIT-TO ( DOCTYPE , "ar" ) ) AND ( LIMIT-TO ( LANGUAGE , "English" ) )

Wos:

TS=("Brain Neoplasms" OR "Neoplasm Recurrence" OR "Glioma" OR "Astrocytoma" OR "Oligodendroglioma" OR "Glioblastoma" OR "Glioma, Subependymal" OR "Optic Nerve Glioma" OR "Neoplasm Metastasis" OR "diagnosis" OR "diagnostic imaging" OR "radiotherapy" OR disease progress* OR true progress* OR recurrent glio* OR residu* OR recurr* OR progress* OR true tumo* OR glioma* OR (brain* AND (tumor OR tumour)) OR glioblastom* OR astrocytom* OR oligodendrogliom* OR oligoastrocytom* OR (glia* AND (tumor OR tumour)) OR brain met*)
AND
TS=("Radiation Induced" OR "Treatment Outcome" OR "radiation effects" OR treatment induc* OR radiation induc* OR radiation associat* OR radiation chang* OR radiation effect* OR treatment necros* OR chemoradiation* OR treatment effect* OR post treat* OR posttreat* OR posttherap* OR post therap* OR postsurg* OR post-surg* OR post irradiat* OR postirradiat* OR after irradia* OR after rad* OR post radiat* OR postradiat* OR treatment outcome* OR radiation injur* OR pseudo progress* OR pseudo response OR pseudoprogress* OR pseudorespon* OR radiation necro* OR radio necro* OR radionecros* OR treatment-relat*)
AND
TS=("Magnetic Resonance Angiography" OR "MR perfusion" OR "MRI perfusion" OR "perfusion-weighted MR" OR "magnetic resonance perfusion" OR "PWI" OR "ASL" OR "arterial spin labeling" OR "DSC" OR "dynamic susceptibility contrast" OR "DCE" OR "Dynamic contrast enhanced" OR "advanced MRI" OR "multiparametric MR" OR "Contrast enhanced MRI" OR "contrast clearance" OR "TRAM" OR "treatment response assessment" OR "delayed contrast MR" OR "delayed contrast extravasation MR")
